# Matrix Metalloproteinase 9 (MMP-9) and Interleukin-8 (IL-8) in Gingival Crevicular Fluid after Minimally Invasive Periodontal Surgery with or without Er:YAG and Nd:YAG Laser Application

**DOI:** 10.3390/antibiotics13080704

**Published:** 2024-07-27

**Authors:** Ewa Dolińska, Anna Skurska, Violetta Dymicka-Piekarska, Robert Milewski, Małgorzata Pietruska

**Affiliations:** 1 Department of Periodontal and Oral Mucosa Diseases, Medical University of Bialystok, ul. Waszyngtona 13, 15-269 Bialystok, Poland; annaskurska@wp.pl (A.S.); mpietruska@wp.pl (M.P.); 2 Department of Integrated Dentistry, Medical University of Bialystok, ul. M. Skłodowskiej-Curie 24A, 15-276 Bialystok, Poland; 3 Department of Clinical Laboratory Diagnostics, Medical University of Bialystok, ul. Waszyngtona 15, 15-269 Bialystok, Poland; violetta.dymicka-piekarska@umb.edu.pl; 4 Department of Biostatistics and Medical Informatics, Medical University of Bialystok, ul. Szpitalna 37, 15-295 Bialystok, Poland; robert.milewski@umb.edu.pl

**Keywords:** periodontal regeneration, interleukin-8, metalloproteinase-9, inflammatory markers, periodontal intrabony defects, Er:YAG laser, Nd:YAG laser

## Abstract

Background: This study aimed to evaluate alterations in the concentrations of matrix metalloproteinase-9 (MMP-9) and interleukin-8 (IL-8) within gingival crevicular fluid (GCF) extracted from the intrabony periodontal defect site before and after minimally invasive regenerative surgery, with or without supplemental laser application. The surgical procedure was performed using the modified minimally invasive surgical technique (M-MIST). Methods: Thirty-eight patients, each presenting with a single vertical defect, were randomly assigned to either the test (M-MIST + Er:YAG + Nd:YAG) or the control group (M-MIST). IL-8 and MMP-9 levels (primary outcomes of the study) were assessed prior to therapy, after 2 and 4 weeks, and 6 months following the surgical procedure by means of dedicated ELISA kits. Results: Both procedures were clinically effective as evidenced by probing depth (PD) reduction and clinical attachment level (CAL) gain at the 6-month follow-up. No statistical differences were observed in the levels of MMP-9 and IL-8 between the groups at any time point assessed. The changes in the level of MMP-9 and IL-8 over time were not statistically significant in any group. IL-8 was positively correlated with MMP-9 in the control group throughout the study and in the test group 2 weeks and 6 months post-op. Conclusions: Within the limitations of this study, the additional application of Er:YAG + Nd:YAG lasers alongside the M-MIST procedure did not enhance the clinical and biochemical treatment outcomes compared to M-MIST alone.

## 1. Introduction

Periodontitis is a multifactorial, infectious disease that leads to the immunologically mediated damage of teeth-supporting tissues and, consequently, tooth loss [1]. The course of periodontitis varies among patients and its advanced forms concern about 10 percent of the population worldwide, which makes it a social disease [2]. The wide spread of the disease means that its treatment generates tremendous costs for healthcare systems worldwide [3].

In some patients, in the course of periodontitis, intrabony defects occur. Their etiology is not fully explained yet. Occlusal trauma, food impaction, or the presence of plaque-retention factors are taken into account in their formation. Attention is also paid to the distance between the roots of adjacent teeth [4]. In the case of root proximity, inflammation affects the entire bone septum between roots. If the bone is wider than the “firing” range of dental plaque, the bone septum is only partially destroyed, which results in the formation of a subcrestal bone defect [5]. The presence of intrabony defects is indicative of stage III and IV periodontitis, thereby exacerbating the prognosis of the disease [6].

The presence of intrabony defects is an indication for regenerative procedures. The most frequently used are guided tissue regeneration (GTR) and biomodification of the root with enamel matrix derivatives (EMDs) or EMD and bone graft [7,8,9]. Nowadays, more and more often, minimally invasive surgery (MIS) in periodontal regeneration is performed. MIS offers the advantage of reducing morbidity, minimizing scars, and mitigating other postsurgical complications in treated patients [10]. In cases of bone defects that spread no more than one-third of the circumference of the tooth, there is a possibility to use an M-MIST (modified minimally invasive surgical technique) surgery, which shows promising results [11]. However, a previous study reported no statistically significant differences in terms of CAL gain and PD reduction when MIS was compared with an extended flap with papilla preservation [12].

Laserotherapy’s importance has also been increasing in recent years. It is used as an adjunct to periodontal treatment [13,14]. The Er:YAG laser has the ability to ablate hard and soft tissues [15]. It can also modify the root surface and remove dental calculus [16,17]. Er:YAG laser application facilitates the adhesion of fibroblasts to the root surface better than scaling and root planing alone [18]. The Nd:YAG laser has different indications. It is used to inactivate microorganisms, to remove the periodontal pocket epithelium, and to improve hemostasis [19]. There are also reports of the combined use of both types of lasers in periodontal non-surgical treatment. The Er:YAG laser enables decontamination of the root surface and hard deposits ablation, and the Nd:YAG laser accelerates clot formation [20,21].

Non-resolving inflammation is a main cause of periodontal hard and soft tissue destruction [22]. Cytokines, matrix metalloproteinases (MMPs), and their inhibitors, growth factors and other regulators in the periodontium, are synthesized in response to bacteria and their products, inducing and maintaining the inflammatory response [23]. IL-8 is a chemoattractant cytokine produced by a variety of tissue and blood cells with a distinct target for recruitment and activation of neutrophils [24]. Neutrophils represent the major inflammatory infiltrate in the periodontium. Neutrophil enzymes released after activation can effectively degrade connective tissue. IL-8 is of considerable interest for a better understanding of the mechanisms leading to neutrophil connecting tissue destruction [25]. Wide range of metalloproteinases (MMPs), enzymes that degrade the extracellular matrix within periodontal tissues. are also involved in this process. MMPs’ expression in mature tissues is normally low, but it rises in pathologies like inflammatory diseases, metastasis, or tumor growth [26]. MMPs are a big family of zinc-dependent endopeptidases and collectively are able to destroy most of the extracellular matrix proteins in the periodontium. Many studies have described interstitial collagenases (collagenases 1, 2, 3, MMP-1, MMP-8, MMP-13), gelatinases A and B (MMP-2, MMP-9) and stromelysin (MMP-3) in gingival crevicular fluid (GCF) [27]. However, interpreting the GCF concentration of specific enzymes is challenging. For instance, enzymes derived from neutrophils, like MMP-8, offer information about the number of these cells in the periodontal pocket rather than the destruction of periodontal tissues. The role of gelatinases in inflammation is also still not clear, and its GCF concentration is hard to interpret [28]. A relationship between MMP-9 GCF concentration and clinical periodontal parameters including attachment loss was noted [29]. It was also found that levels of MMP-9 are increased in patients with periodontitis and decrease after periodontal therapy [30]. MMP-9 is involved not only in the soft tissue breakdown, but there are scarce reports of its indirect role in immune-mediated bone loss in the periodontium [31,32], so MMP-9 may play a role in the mediating of the bone defects’ formation.

Given the aforementioned considerations, the objective of our study was to assess changes in the levels of MMP-9 and IL-8 in periodontal pockets corresponding to intrabony periodontal defects eligible for regenerative procedures, specifically M-MIST alone or M-MIST with additional Er:YAG and Nd:YAG laser applications, over a six-month follow-up period. We assumed changes in immunological parameters (MMP-9, IL-8) as the primary endpoints and clinical parameters, such as PD reduction and CAL gain, as the secondary endpoints of the study. We formulated the null hypothesis that additional laser usage does not affect MMP-9 and IL-8 levels, nor does it affect the values of clinical parameters.

## 2. Results

All 38 patients with periodontitis (stage III) [33] completed the 6-month follow-up visits with no further dropouts and a 100% retention rate. No adverse events were reported. Patients were equally distributed between the study and control groups without differences according to age, gender, or intrasurgical defect depth. Table 1 depicts the characteristics of patients, with teeth and defects included.

Included patients were characterized by good oral hygiene expressed by the FMPS (full mouth plaque score) index [34]. At baseline, there was no person with an FMPS over 20%. The mean FMPS in the test group amounted to 10.8%, and it was 11.6% in the control group. FMBOP (full mouth bleeding on probing) was also low [35]. It was 10.7% in the test group and 10.3% in the control group, respectively.

**Figure 1:**
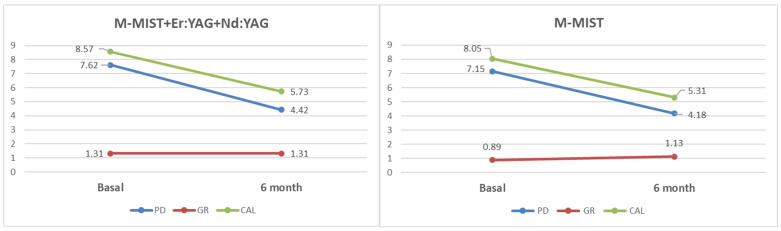
Mean changes in clinical parameters at the six-month follow-up in the test and control groups.

Clinical parameters of the operated area are presented in Table 2 and Figure 1. In both groups, there were significant PD reduction and CAL gain in the 6-month observation period. Gingival recession (GR) increased in the control group and did not change in the test group. The changes in GR were not significant over time. There were also no significant differences between both groups studied before or six months post-op.

**Figure 2:**
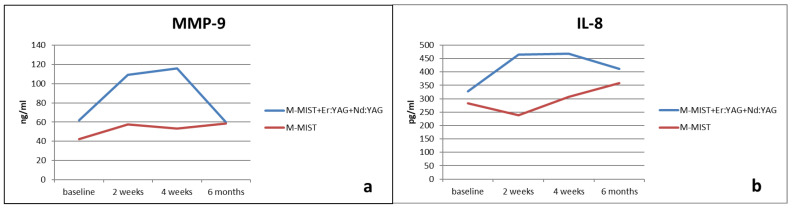
(**a**) Mean changes in the MMP-9 amount at the six-month follow-up. (**b**) Mean changes in the IL-8 amount at the six-month follow-up.

Changes in MMP-9 levels (Table 3, Figure 2a) obtained from periodontal pockets that underwent surgical treatment were not significant in any group at the six-month follow-up. We did not note differences between groups either. However, after Er:YAG and Nd:YAG laser application, the MMP-9 amount increased much more in the test than in the control group after 2 and 4 weeks (without significance).

Similar results for the IL-8 amount were noted. There were no significant differences between groups or at the 6-month follow-up in any group. But, the most visible changes in IL-8 levels were noted 2 and 4 weeks after Er:YAG and Nd:YAG laser surgery, although this was without significance. Mean changes in IL-8 levels are depicted in Table 4 and Figure 2b.

**Figure 3:**
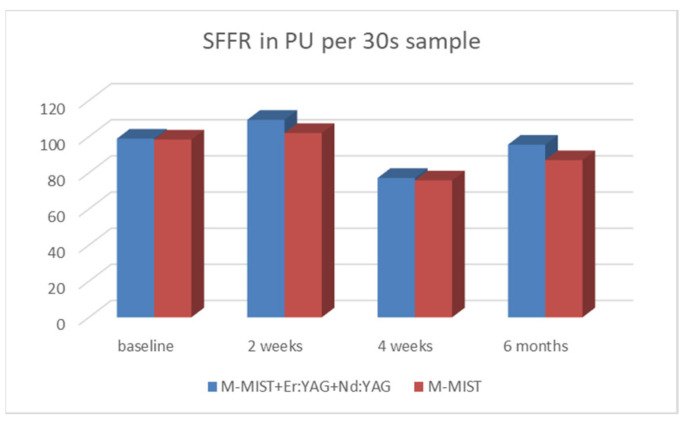
Mean changes in the SFFR volume in the 30 s sample at the six-month follow-up in the test and control groups.

The GCF volume (SFFR, sulcus fluid flow rate) expressed in relative PU did not change over time in the test group, but it was on the verge of significance in the control group during the six-month observation period. There were no differences between groups. The SFFR in both groups slightly increased after a week following post-surgical intervention to increase after 2 weeks and finally decrease after 6 months. Mean changes in the SFFR are shown in Table 5 and Figure 3.

The most important correlation noted was the moderate dependence of IL-8 and MMP-9. The amount of both molecules correlated at every time point in the control group. Similar correlations were observed in the test group, but only after 2 weeks and 6 months. Many other correlations were checked. MMP-9 correlated with SFFR after 4 weeks and 6 months, and MMP-9 correlated with PD after six months in the control group. MMP-9 and SFFR correlated in the test group only after 6 months. Spearman rank order correlations are shown in Table 6 and Table 7.

## 3. Discussion

The results of the present study have shown that the effectiveness of intrabony defect treatment with M-MIST or M-MIST in combination with Er:YAG + Nd:YAG lasers is similar. Both methods were equally effective and led to statistically significant improvements in clinical parameters. PD reduction and CAL gain were almost the same in both groups, with a slight difference in favor of the M-MIST group (without significance). Healing was uneventful in both groups, and no adverse events were noted. This proves that the procedures performed were not invasive and the post-treatment healing was good. The results that plaque and bleeding indices were low indicate proper hygienic preparation of the patients and an optimal level of plaque control before reconstructive surgery, which is essential for undisturbed healing. Another important factor influencing the response to periodontal surgical treatment is smoking. Smoking has a negative effect on bone healing and regeneration [36]. That is why smokers were not included in the study.

In the case of the coexistence of residual pockets with vertical defects, periodontal reconstructive surgery procedures are always considered [37]. Recognized factors that also influence the effect of surgery include obtaining appropriate decontamination of the treatment area. Additional laser usage as a new solution may be a valuable option in the standard surgical procedure. By eliminating calculus and bacteria, Erbium-Doped Yttrium Aluminium Garnet (Er:YAG) and Neodymium-Doped Yttrium Aluminum Garnet (Nd:YAG) lasers can create a biocompatible root surface that may facilitate periodontal healing [38]. Due to different features, these two lasers have different effects on the hard and soft tissues. However, it should be taken into consideration that the use of lasers in periodontal treatment gives ambiguous results [14]. There are not many papers published evaluating the combined utilization of Er:YAG + Nd:YAG lasers in oral surgery. The available literature mostly refers to the use of Er:YAG + Nd:YAG in non-surgical periodontal treatment, where such a combination of lasers may lead to additional clinical improvements compared to non-surgical treatment alone [21].

Clinical examination is a gold standard to evaluate the effectiveness of periodontal reconstructive procedures, but knowledge about the changes that occur at the molecular level in the treated site are of most interest. Not much is still known about gingival crevicular fluid (GCF) immunological changes in intrabony defects after regenerative procedures [39]. We assessed the levels of IL-8 and MMP-9 in GCF obtained from the periodontal pockets corresponding to vertical defects subjected to M-MIST or M-MIST with additional laser utilization.

GCF is an altered serum transudate/inflammatory exudate. In healthy tissues, GCF originates from blood vessels, and it is produced thanks to the osmotic gradient. However, leukocytic infiltrate and polymorphonuclear leukocytes are always present in the junctional epithelium and in the sulcus. Numerous cytokines and enzymes are released to GCF, including host response factors, molecules from blood, local tissues, and plaque bacteria [40]. The fluid is easily collected, so it is a valuable source of locally and systematically derived biomarkers. It is at the center of contemporary research projects because useful diagnostic biological markers are researched to detect subclinical alterations in tissue metabolism before clinically visible damage can occur. As it is known, not only the composition but also the volume and flow rate of GCF are of importance. The relationship between the enlarged volume of GCF and the increased severity of inflammation has been well-documented [41,42]. In our study, the sulcus flowing flow rate (SFFR) increased at 2 weeks after surgery to decrease at 4 weeks and thereafter come back to baseline levels at 6 months. This is in line with the observations of other authors, who observed a rise in the volume of GCF shortly after surgery (0–2 weeks) and thereafter a volume decrease [43,44]. In previous work, our group investigated molecular changes in GCF after periodontal regenerative procedures, and we also noticed a rise in the SFFR one week after the surgery, and it remained high 2 weeks following the procedure. This post-surgical increase in GCF volume may indicate the enhancement of inflammation during early surgical wound healing [45].

Intrabony periodontal defects may differ from healthy sites in terms of the molecular GCF profile. This profile may also be changed by periodontal treatment. Not much is known about such changes, but the first studies trying to find immunological descriptions of intrabony defects have been recently published. In the clinical study comparing the molecular profile of intrabony defects with healthy sites utilizing a multiplex bead immunoassay, 27 markers were assessed. Sites with an intrabony defect presented increased IL-1α, IL-1β, IL-6, INFγ, and MMP-8 levels compared with healthy sites. Additionally, FGF and VEGF levels were elevated, and traits of cell aging were observed [46]. The same group examined the molecular profile of periodontal pockets corresponding to intrabony defects after minimally invasive non-surgical periodontal therapy (MINST) [47]. The authors focused on changes after 1 and 5 days and 3 months after MINST. The most pronounced molecular changes were observed a day after treatment. It returned to baseline after 3 months. Levels of IL-2, IL-4, IL-8, MMP-1, MMP-3, TIMP-1, and FGFb had significantly risen in GCF one day after non-surgical periodontal therapy. 

Not many scientific studies refer to immunological parameters of surgically treated sites in the periodontium. Pellegrini et al. assessed wound healing proteins in GCF after regenerative and open flap debridement procedures. They concluded that MMP-1 and bone morphogenetic protein-7 were associated with periodontal regeneration because of the increase in its levels in patients who responded well to therapy [48]. Accelerated healing of infrabony defects after enamel matrix derivative application expressed by a rapid return to baseline of TIMP-1, MMP-1, and MMP-8 levels was observed by Okuda et al. [44]. Other authors found transforming growth factor-β1 useful for monitoring periodontal repair and regeneration [43] or the expression of growth mediators in GCF of patients after periodontal surgery [49].

We opted to evaluate MMP-9 and IL-8 due to existing evidence in the literature suggesting that these molecules may play a role not only in the destruction of soft tissues but also in bone resorption and remodeling. MMPs are proteases involved in extracellular matrix destruction in periodontitis. MMP-9 is responsible for the degradation of many proteins, such as basement membrane collagen and laminin [28]. Elevated levels of MMP-9 were observed in periodontitis patients compared with healthy subjects. It was demonstrated that periodontal treatment reduced the MMP-9 level dramatically [50,51]. There are many scientific studies assessing the collagenolytic role of MMPs, but there is still a scarcity of data from studying MMPs as regulators of periodontal inflammation [51]. It was proven that MMP-9 along with MMP-13 are involved in alveolar bone resorption and periodontal tissue destruction [52,53]. MMPs may not only degrade bone collagen matrix but also may indirectly modulate bone resorption via osteoclast activation. The mechanisms include the activation of osteoclast-secreted proMMP-9, which denaturates collagen derived from MMP-13 activity. The other is cleaving galectin-3, which is an inhibitor of osteoclastogenesis that nullifies its inhibitory effect. The next is regulating the receptor activator of nuclear factor-κB ligand (RANKL)/osteoprotegrin axis in favor of RANKL [31,51]. Taking into consideration the above, it can be assumed that MMP-9 is a regulator of periodontal bone lesions. We observed no significant differences between control and study groups according to MMP-9 levels. There were also no intragroup changes over time according to gelatinase B. However, in both groups, early after surgery (2 and 4 weeks), the amount of MMP-9 had risen. This change was more pronounced in the study group, but it was not significant. In the test group, there was a sharp increase, and, evidently, this change was more pronounced in this group but it was not significant. Interestingly, after 6 months, in both groups the level of MMP-9 came back to the value that was near the baseline level, and it was almost the same in both groups. This may indicate an increase in the release of MMP-9 after laser stimulation. Unfortunately, this cannot be confirmed because no study assessed MMP-9 after surgical therapy with additional laser usage.

Sijari et al. assessed IL-8 after resective periodontal surgery. They observed a decrease in its levels after therapy along with better early healing [54]. This is partially consistent with our results, because in the M-MIST group after 2 weeks, the amount of IL-8 was decreased, but then it rose to be higher than baseline levels. In the test group, we noticed an increase in IL-8 at all time points examined. Our results were not significant. There were no differences between groups either. What is worth noting after laser usage is that there was an increase in the IL-8 level in the test group, and there was a horizontal trend in its high concentration lasting 2 weeks. Then, after 6 months, the IL-8 level slightly decreased. In contrast, in the control group, right after surgery the IL-8 amount decreased, and after 6 months it reached higher than baseline levels. Despite the lack of statistical differences, this proves that laser therapy has an impact on the IL-8 level.

IL-8 (CXCL8) is a strong chemoattractant for neutrophils, and it stimulates fast degranulation of gelatinase B. MMP-9 cleaves IL-8 to more potent chemoattractants. It results in a positive feedback loop for neutrophil activation and chemotaxis and in the increased influx of neutrophils to fight infections [55]. In our study, the level of IL-8 correlated positively with MMP-9 at all time points in the control group and after 2 weeks and 6 months in the study group. The strength of correlations was moderate. It is interesting that despite the many parameters checked we recorded only single additional relationships between them, i.e., MMP-9 correlated positively with SFFR after 6 months in both groups. Our research group observed a similar positive correlation between IL-8 and MMP-9 after guided tissue regeneration with and without systemic antibiotics throughout the study [45].

Our study is well-planned and meticulously conducted research, but it has some drawbacks. A major limitation was the mediocre clinical sample size. This sample size was calculated on the basis of trials investigating clinical parameters after periodontal reconstructive surgery. Recruiting more patients would allow for a larger sample for biochemical evaluation. A weak point as well is the lack of reference values for immunological parameters, as the cytokine profile after different treatment strategies is only investigated. There is still a lack of knowledge of many molecular processes occurring in the periodontium in the state of inflammatory disease and after surgical and non-surgical therapies. Another aspect is not including smokers, and, therefore, these results should be taken with caution as smokers will undergo these kinds of clinical interventions.

Innovative procedures very often bring additional benefits and become standard treatment options. However, this is not always the case. In our study, we fail to find any advantages of laser-assisted M-MIST surgery. Neither clinical nor molecular results confirmed the superiority of the Er:YAG or Nd:YAG procedure. However, more clinical studies should be conducted to explore the potential of Er:YAG and Nd:YAG lasers in periodontal surgical therapy. The role of MMP-9 and IL-8 in periodontal bone resorption is also unclear. More in vitro and in vivo studies are recommended to fully elucidate direct and indirect actions of both molecules.

## 4. Materials and Methods

### 4.1. Study Population and Experimental Design

The study was designed as a single-center, randomized, prospective, controlled clinical trial. It was performed according to the Helsinki Declaration after previous acceptance obtained from the local bioethical committee (Bioethical Committee, Medical University of Bialystok, Poland R-I-002-397-2016). Each patient entering this research project signed an informed consent form. Thirty-nine generally healthy adults diagnosed with stage III periodontitis [33] were enrolled in the study. Thirty-eight (aged 24–73, mean age 45.4) were analyzed because of one patient’s resignation. Among the analyzed participants, there were 21 women and 17 men. 

The inclusion criteria were as follows:

- Presence of an intrabony defect, with a pocket depth (PD) ≥ 6 mm and a radiological defect depth of ≥3 mm and width of ≥2 mm;
- Over 18 years of age;
- Full mouth plaque index (FMPI) < 20% and full mouth bleeding on probing (FMBOP) < 20% [34,35].

Patients with general diseases that could affect the healing process, smokers, as well as pregnant or breastfeeding women were excluded.

Allocation of patients to test (M-MIST + Er:YAG + Nd:YAG) and control (M-MIST) groups was performed through a coin toss just before the surgery. Each participant had only one intrabony defect treated in the project. The patient and the surgeon were not blinded in the protocol because of technical reasons.

### 4.2. Clinical Examinations, Surgery, and Postoperative Care

The following clinical parameters were measured before surgery and 6 months post-op for each tooth with an intrabony defect: probing depth (PD), gingival recession (GR), and clinical attachment level (CAL). Each tooth was probed at six points (mesial, middle, and distal on both buccal and lingual sides). The cemento-enamel junction (CEJ) or the filling margin were taken as the reference points.

The full mouth plaque score (FMPS) and full mouth bleeding on probing (FMBOP) were calculated as a percentage based on the four surfaces of each tooth.

All measurements were performed with the use of a periodontal probe (PCP UNC15, Hu-Friedy, Chicago, IL, USA) and taken by the same experienced and calibrated examiner.

Intraoral radiographs were captured at baseline and after 6 months. To guarantee precision, a long cone parallel technique positioner was custom-prepared for each of the enrolled patients.

All surgical interventions were performed under local anesthesia (Septanest 100, Septodont, Paris, France). The surgical procedure consisted of intrasulcular incisions and preparation of the mucoperiosteal flap according to the principles of papilla preservation [56,57] and minimally invasive techniques [58].

In the test group, additionaly to modified minimally invasive surgery (M-MIST) dental laser (Fotona Light Walker AT-S, Dallas, TX, USA) was used.The granulation tissue was excised using the Er:YAG laser with parameters set at 3 W, 150 mJ, and 20 Hz LP, while the debridement of the root surface was accomplished with the same Er:YAG laser, operating at 1.6 W, 160 mJ, and 10 Hz LP. Concluding the surgical procedure, the formation of a blood clot was induced utilizing the Nd:YAG laser, configured at 2 W and 20 Hz VLP.

In the control group, the elimination of granulation tissue and the scaling and planing of root surfaces were carried out using manual instruments, specifically Gracey curettes (Hu-Friedy, Chicago, IL, USA) and ultrasonic scalers (EMS Piezon Tip PS, EMS, Nyon, Switzerland).

Once the debridement of the intrabony defect was completed, the mucoperiosteal flap was repositioned and stabilized by means of vertical modified mattress sutures (Ethilon 5.0, Johnson & Johnson Company, New Brunswick, NJ, USA).

Tooth mobility was an indication of splinting.

After surgery, patients were instructed to rinse the mouth twice daily using 0.2% chlorhexidine solution (Eludril, Pierre Fabre Laboratories, Paris, France), refrain from eating hard food, and avoid vigorous tooth brushing at the surgical area for 2 weeks. The sutures were removed 2 weeks post-op. Check-up appointments were scheduled for 1, 2, and 4 weeks and then at 3 and 6 months. Healing and possible complications (flap dehiscence, flap or papillae necrosis, suppuration, inflammation, as well as pain exacerbations) were monitored during the follow-up appointments. Additionally, at check-ups, the supragingival plaque was removed, and photographs of the surgical area were taken at every visit.

### 4.3. GCF Sampling

From the periodontal pocket corresponding to the operated intrabony defect, GCF was collected to determine the sulcus fluid flow rate (SFFR) in relative Periotron-units (PU) and to investigate IL-8 and MMP-9 levels. After the isolation of the tooth with cotton rolls and air-drying, the visible dental plaque was removed. Then, paper strips (Periopaper, Interstate Drug Exchange, Amityville, NY, USA) were placed in the periodontal pocket at a 1–2 mm depth for 30 s. The blood-contaminated strips were thrown away. The GCF volume absorbed on a paper strip (SFFR) was measured using a calibrated device (Periotron 8010, Oraflow, Plainview, NY, USA) and expressed in Periotron Units (PU). After measurement, the samples were immediately placed in Eppendorf tubes containing 200 μL of phosphate-buffered saline (PBS) and frozen.

### 4.4. GCF IL-8 and MMP-9 Analysis

The gingival crevicular fluid (GCF) samples collected at baseline and after 2 weeks, 4 weeks, and 6 months were used for laboratory analysis. The concentrations of IL-8 and MMP-9 in GCF were determined with the use of commercially available ELISA kits (Human CXCL8/IL8, R&D Systems, Minneapolis MN, USA and Human MMP-9 Elisa kit, R&D Systems, Minneapolis, MN, USA) following the manufacturer’s instructions. The results were presented as the amount per 30 s per measurement point and expressed in pg/mL for IL-8 and ng/mL for MMP-9. We reported the total mediator content per 30 s sample because it removes a potential source of error from the analysis [59] and it is widely used in GCF elaborations [60].

### 4.5. Statistical Analysis

In the statistical analysis, the normality of distribution was verified using the Shapiro –Wilk test and the Kolgomorov–Smirnov tests with Lillefors correction. The Wilcoxon pair test was used to compare dependent variables over time. The nonparametric U Mann–Whitney test was used to compare the quantitative independent variables without distribution normality. ANOVA Friedman’s test with Kendall’s coefficient was used for multiple comparisons. Spearman’s rank correlation coefficient was also determined. 

The results were considered statistically significant for *p* < 0.05. Statistica 13.3 (TIBCO Software Inc. Palo Alto, CA, USA) was employed for calculations.

The sample size was calculated a priori by making an assumption about a standard deviation of a CAL change of 1 mm and to detect a mean difference of 1 mm with a test power of 80% on 32 subjects. However, considering possible drop-outs, 39 patients were recruited and randomized to the study.

## 5. Conclusions

Within the limits of our study, the results indicate that the additional use of Er:YAG + Nd:YAG lasers with the M-MIST procedure does not improve the clinical and biochemical treatment outcomes compared to M-MIST alone.

## Figures and Tables

**Table 1:** Characteristics of study participants and teeth included in the surgical procedures (clinical parameters in mm).

	Test (M-MIST + Er:YAG + Nd:YAG)	Control (M-MIST)	Significance
Number of patients	19	19	-
Gender	12F/7M	9F/10M	NS (*p* * = 0.34)
Age (range)	47 (30–73)	43.5 (24–59)	NS (*p* * = 0.9)
Incisors/canines/premolars/molars	3/3/12/1	3/1/9/6	-
Mean intrasurgical defect depth	4.03 ± 1.58	4.34 ± 1.13	NS (*p* * = 0.14)
FMPS (%)	10.8 ± 4.42	11.6 ± 4.68	NS (*p* * = 0.53)
FMBOP (%)	10.71 ± 3.89	10.34 ± 4.58	NS (*p* * = 0.83)

*p* *—U Mann–Whitney test.

**Table 2:** Clinical parameters in test (M-MIST + Er:YAG) and control (M-MIST) groups at baseline and 6 months post-op.

	PD (mm)			GR (mm)			CAL (mm)		
	M-MIST + Er:YAG + Nd:YAG	M-MIST	*p* **	M-MIST + Er:YAG + Nd:YAG	M-MIST	*p* **	M-MIST + Er:YAG + Nd:YAG	M-MIST	*p* **
Baseline (mean)	7.62 ± 1.44	7.15 ± 1.25		1.31 ± 1.34	0.89 ± 1.19		8.57 ± 2.16	8.05 ± 1.8	
6 months (mean)	4.42 ± 1.30	4.18 ± 1.14	NS	1.31 ± 1.00	1.13 ± 1.35	NS	5.73 ± 1.48	5.31 ± 2.02	NS
	*p* * = 0.0001	*p* * = 0.0002		NS	NS		*p* * = 0.0002	*p* * = 0.0003	
Baseline (median)	7 (6–11)	7 (5–9)		1.5 (0–4)	0 (0–4)		8 (5–15)	8 (5–12)	
6 months (median)	4 (3–7)	4 (3–7)		1 (0–3)	1 (0–3)		6 (4–9)	5 (3–10)	
Diff.	2.84 ± 0.96	2.97 ± 1.18	NS	0.00 ± 0.86	−0.24 ± 0.79	NS	2.84 ± 1.45	2.74 ± 1.45	NS

p *—Wilcoxon pair test (changes in time); *p* **—U Mann–Whitney test (between groups); NS—non-significant; Diff.—difference 0–6.

**Table 3:** Mean changes in the MMP-9 amount at the six-month follow-up in the test (M-MIST + Er:YAG + Nd:YAG) and control (M-MIST) groups expressed as ng/mL per 30 s sample.

MMP-9			
	M-MIST + Er:YAG + Nd:YAG	M-MIST	*p* ** (Between Groups)
Baseline	61.96 ± 87.86	42.32 ± 30.52	NS
2 weeks	109.04 ± 207.35	57.51 ± 65.86	NS
4 weeks	115.96 ± 125.99	53.34 ± 46.71	NS
6 months	59.78 ± 59.45	58.57 ± 49.44	NS
*p* * (changes in time)	NS	NS	
Diff. 0–2 w	−47.08 ± 187.36	−15.19 ± 70.34	NS
Diff. 0–4 w	−54 ± 151.00	−11.02 ± 54.02	NS
Diff. 0–6 m	2.18 ± 89.74	−16.25 ± 52.14	NS

*p* *—Anova Friedman’s for multiple comparisons; *p* **—U Mann–Whitney test; NS—non-significant, Diff.—difference.

**Table 4:** Mean changes in the IL-8 amount at the six-month follow-up in the test (M-MIST + Er:YAG + Nd:YAG) and control (M-MIST) groups expressed as pg/mL per 30 s sample.

IL-8			
	M-MIST + Er:YAG + Nd:YAG	M-MIST	*p* ** (Between Groups)
Baseline	327.22 ± 281.55	283.20 ± 158.41	NS
2 weeks	464.84 ± 477.98	238.03 ± 141.13	NS
4 weeks	467.91 ± 313.12	307.77 ± 256.33	NS
6 months	410.97 ± 300.62	357.77 ± 229.54	NS
*p* * (changes in time)	NS	NS	
Diff. 0–2 w	−137.62± 448.06	45.17± 182.89	NS
Diff. 0–4 w	−140.69± 387.93	−24.57± 311.88	NS
Diff. 0–6 m	−83.75 ± 341.86	−74.56 ± 285.53	NS

*p* *—Anova Friedman’s for multiple comparisons; *p* **—U Mann–Whitney test; NS—non-significant, Diff.—difference.

**Table 5:** Mean changes in the SFFR volume expressed in the relative PU at the six-month follow-up in the test (M-MIST + Er:YAG + Nd:YAG) and control (M-MIST) groups per 30 s sample.

SFFR			
	M-MIST + Er:YAG + Nd:YAG	M-MIST	*p* ** (Between Groups)
Baseline	98.89 ± 30.14	98.42 ± 43.21	NS
2 weeks	109.31 ± 33.65	102.00 ± 37.08	NS
4 weeks	77.15 ± 28.67	75.78 ± 30.75	NS
6 months	95.57 ± 46.96	87.00 ± 35.46	NS
*p* * (changes in time)	NS	*p* = 0.046	
Diff. 0–2 w	−10.42 ± 53.56	−3.58 ± 53.40	NS
Diff. 0–4 w	21.74± 37.33	22.64 ± 46.15	NS
Diff. 0–6 m	3.32 ± 43.13	11.42 ± 51.02	NS

*p* *—Anova Friedman’s for multiple comparisons; *p* **—U Mann–Whitney test; NS—non-significant, Diff.—difference.

**Table 6:** Correlations (Spearman test) between GCF IL-8 and MMP-9 levels at the 6-month follow-up in the test (M-MIST + Er:YAG + Nd:YAG) and control (M-MIST) groups in the operated region.

IL-8 and MMP-9	M-MIST + Er:YAG + Nd:YAG		M-MIST	
	R	*p*	R	*p*
Baseline	-	NS	0.62	0.0045
2 weeks	0.68	0.0015	0.74	0.0003
4 weeks	-	NS	0.59	0.0082
6 months	0.58	0.0087	0.67	0.0016
NS—non-significant				

**Table 7:** Other investigated correlations (Spearman test) in the test (M-MIST + Er:YAG + Nd:YAG) and control (M-MIST) groups.

	Correlation	M-MIST + Er:YAG + Nd:YAG	M-MIST
Baseline	IL-8 and PD	NS	NS
Baseline	IL-8 and SSFR	NS	NS
Baseline	IL-8 and intra-defect depth	NS	NS
Baseline	MMP-9 and PD	NS	NS
Baseline	MMP-9 and SSFR	NS	NS
Baseline	MMP-9 and intra-defect depth	NS	NS
2 weeks	IL-8 and SFFR	NS	NS
2 weeks	MMP-9 and SFFR	NS	NS
4 weeks	IL-8 and SFFR	NS	NS
4 weeks	MMP-9 and SFFR	NS	R = 0.5, *p* = 0.03
6 months	IL-8 and PD	NS	NS
6 months	IL-8 and SSFR	NS	NS
6 months	MMP-9 and PD	NS	R = 0.5, *p* = 0.026
6 months	MMP-9 and SSFR	R = 0.46, *p* = 0.047	R = 0.5, *p* = 0.029
NS—non-significant			

## Data Availability

The datasets used and/or analyzed during the current study are available from the corresponding author upon reasonable request.
